# Difficult Internal Jugular Central Venous Cannulation Using J-Tip Guidewire with Indwelling Peripherally Inserted Central Venous Catheters

**DOI:** 10.1155/2019/5134575

**Published:** 2019-07-22

**Authors:** Preeti Anand, Minal Joshi, Khaja Ahmed, Joel Yarmush

**Affiliations:** New York Presbyterian Brooklyn Methodist Hospital, USA

## Abstract

Central venous cannulation is a commonly performed procedure while managing critically ill patients; increasingly we encounter patients with indwelling wires or devices, like pacemakers, implantable cardioverter defibrillator devices, and peripherally inserted central venous catheters which complicate insertion of central venous catheters further. We present two cases where use of standard J-tip guidewire may have exacerbated the difficulty associated with internal jugular cannulation in presence of peripherally inserted central venous catheters. Recognition and avoidance of possible complications are crucial, and we discuss complexity posed by indwelling peripherally inserted central venous catheters and possible solutions.

## 1. Introduction

Peripherally inserted central venous catheters (PICC) were first described in 1975, as an alternative to classical central venous catheter (CVC) without the risk of pneumothorax associated with CVC, mainly internal jugular and subclavian venous cannulations. Also, PICC is preferred in conditions requiring extended antibiotic therapy, parenteral nutrition, chemotherapy, accessed through either cephalic or basilic veins in either of upper extremities, with distal tip at superior vena cava (SVC), right atrium junction. PICC lines although perfect for prolonged therapy are not without complications and are inadequate for resuscitative needs in critically ill patients, such as administration of fluid bolus, rapid transfusion, concomitant administration of vasopressors, temporary hemodialysis, central venous pressure monitoring, pulmonary artery catheterization, or transvenous pacing, which necessitate CVC [1]. PICC can potentially obstruct advancement of J-tip guidewire introduced via internal jugular approach which is an essential step in achieving CVC. Although difficulty posed by indwelling venous devices has been documented, there are no case reports or studies to describe or quantify the problem [2].

Authors confirm that patient(s) or patient's family have provided written HIPAA authorization to publish this case report.

## 2. Description

### 2.1. Case 1

**Figure 1:**
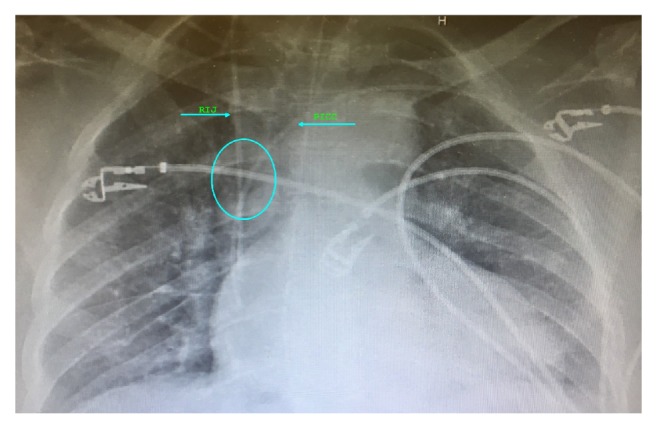
Chest X-ray showing multiple superficial EKG electrodes, left upper extremity PICC line with RIJ central venous catheter optimally positioned at right atrium and SVC junction, importantly with 45-degree angle at convergence.

**Figure 2:**
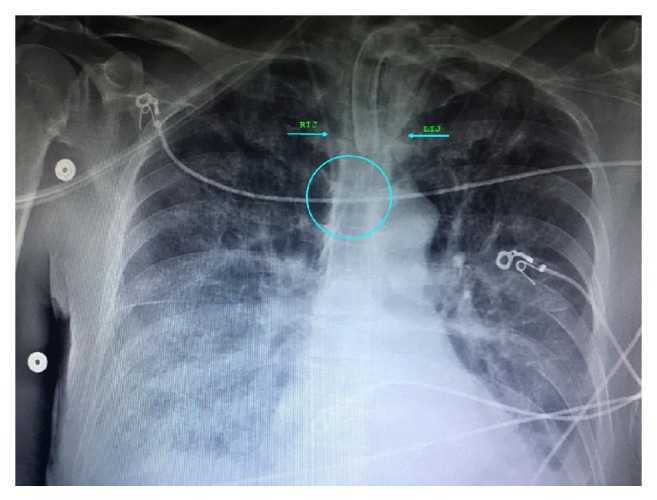
Chest X-ray showing tracheostomy tube, superficial EKG electrodes, and two central venous catheters via left and right internal jugular access in proximity almost parallel position in SVC with 20-degree angle at convergence.

61-year-old female patient with multiple comorbidities, including diabetes mellitus, hypertension, and diverticulitis, presented with pelvic abscess, small bowel obstruction, and sepsis, requiring emergent exploratory laparotomy. She had left upper extremity single lumen PICC, placed two weeks ago for antibiotic therapy which was now inadequate for her resuscitation needs, vasopressors, and central venous pressure monitoring. Right internal jugular (RIJ) vein cannulation was considered due to its straight course and ease of insertion. PICC was not removed before attempting central venous cannulation as that was our only venous access. We used standard Arrow® 7 French triple lumen Kit with soft spring J-tip guidewire, introducer needle puncture was guided by peripheral vascular ultrasound, vascular anatomy was found to be normal, needle placement was uneventful with free back flow, j-tip guidewire was introduced using Seldinger technique, wire position was confirmed with ultrasound, guidewire was advanced smoothly to about 15 cm at the needle hub, 9 cm into the vessel after which it encountered resistance, and we tried manipulating guidewire by changing angle of insertion, but it was not possible to advance further. Hence, left internal jugular (LIJ) vein cannulation was attempted, which was also met with resistance to guidewire insertion approximately 17 cm at needle hub or 11 cm into the vessel, and right internal jugular access was successfully cannulated at 2nd attempt with manipulation of guidewire. Patient did not have history of previous internal jugular cannulations, thoracic surgery, or any other condition predisposing to central venous stenosis or thrombosis. Postprocedure chest X-ray was obtained, which shows CVC in appropriate position, in proximity with PICC, with approximate 45-degree angle at convergence of two catheters and no evidence of complications (Figure 1). PICC was removed after radiological confirmation of optimal CVC placement. Patient remained critical in ICU for a period of two weeks and needed change of CVC for suspected insertion site infection. Left internal jugular CVC was placed without difficulty, complications, or resistance with guidewire insertion, using Arrow® 7 French triple lumen kit and ultrasound guidance for introducer needle puncture. Postprocedure chest X-ray confirmed optimal placement, with approximate 20-degree angle at convergence of two catheters (Figure 2).

### 2.2. Case 2

**Figure 3:**
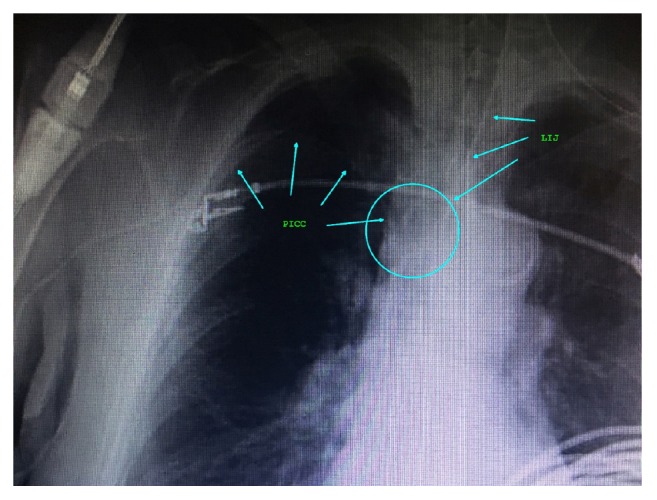
Chest X-ray showing multiple superficial EKG electrodes, endotracheal tube, and right upper extremity PICC line abutting LIJ venous catheter in SVC with 60-degree angle at convergence.

85-year-old female patient with no past medical history except digital osteomyelitis for which she was on intravenous antibiotics via right upper extremity single lumen PICC presented with bowel perforation for which she needed emergent exploratory laparotomy. She was hypotensive and had new onset atrial fibrillation with rapid ventricular rate; vasopressors were started using PICC, but it was essential to have better central venous access for monitoring, vasopressors, and resuscitation considering possibility of septic shock in setting of major arrhythmia. Ultrasound guided RIJ vein cannulation was attempted using 7fr® Arrow multilumen CVC kit with soft spring J-tip guidewire, with good anatomical visualization, and needle puncture, and j-tip guidewire advancement was met with resistance at about 10cm, and it was not possible to advance it further even with guidewire manipulation; guidewire and needle were withdrawn and attempt was made with slightly different puncture site with the same results. Finally, LIJ vein cannulation was achieved on second attempt after being met with resistance the first time. Postprocedure chest X-ray revealed left internal jugular catheter to be well positioned at junction of SVC and right atrium, adjacent to PICC, with no evidence of complications (Figure 3). All the attempted and successful internal jugular cannulations in both cases were done by experienced anesthesiologists and intensivists, given the critical condition of patients.

## 3. Discussion

CVC is most commonly performed by Seldinger technique which essentially means passing catheter over a guidewire [3, 4]. Literature advocates use of J-tip guidewires as they are considered atraumatic with least likelihood of complications due to their shape [5, 6].

In both cases J-tip guidewire introduction into SVC was met with resistance possibly caused by in situ PICC, and it was not possible to cannulate internal jugular from the same side as the PICC. Both of these patients did not have history of previous central venous cannulation which could predispose to central venous stenosis or thrombosis, causing resistance to passage of guidewire.

If we consider venous anatomy, internal diameter of SVC is 15 +/- 3 mm, and right internal jugular and left subclavian approaches veins, respectively, take straight and gently curving trajectories to SVC; however, right subclavian vein takes a near right angle turn into SVC and left internal jugular approach incorporates two turns: one into brachiocephalic vein and second into SVC [7]. These turns create potential for resistance to guidewire placement and venous side wall puncture if excessive force is used in event of failure to negotiate the curve appropriately [8].

**Figure 4:**
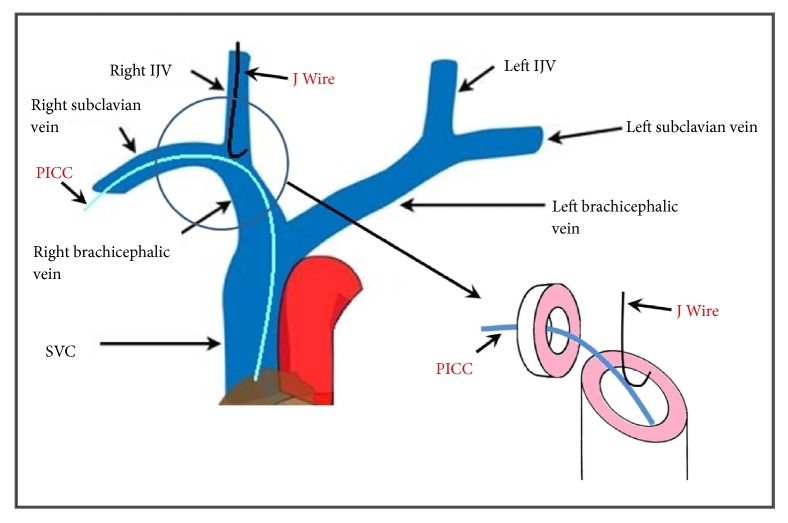
Representation of CVC guidewire and possible PICC interaction. Indwelling PICC in right subclavian encountered by J-tip guide in Right Internal Jugular Vein (IJV).

It is worth noting that SVC with internal diameter of 15+/- 3mm thus bisected by a PICC would leave 9mm on either side. A 4F-PICC has outer diameter of 1.35mm and 6F with outer diameter of 2mm further reduces space on either side by 0.7 to 1mm, leaving 8 to 8.5 mm at maximum to negotiate a J-tip guidewire. J-tip has a diameter of 10mm, making this an extremely challenging task to achieve this without complications when angle at convergence point is greater than 60 degrees, while it is much easier when guidewire is oriented more parallel to the indwelling catheter, as demonstrated by both of our cases. Ipsilateral subclavian and internal jugular veins converge at near 90-degree angles explaining difficulty encountered while cannulating internal jugular vein with indwelling catheter entering SVC through the same side subclavian vein (Figure 4).

Difficulty in CVC with permanent implanted pacemaker is well established. They most commonly have lead size of 4F to 6F and use subclavian or cephalic vein approach. PICC have similar size and vascular approach and thus behave like a pacemaker lead in situ.

It is essential to avoid force while advancing guidewire when met with resistance, to prevent potential injury to vascular structures or cardiac chamber. Kinking, looping, knotting, and fracture of guidewire resulting in embolization and cardiac arrest have been reported [5, 6, 9–13].

Safe use of guidewire requires care in handling and understanding of its physical characteristics [9, 11, 12]. J shaped end of guidewire results from rounding and flattening of core which causes structural weakness and may lead to potential breakage if excessive force is used [9, 11, 12].

Although guidewires with straight tip and angle tip are available to interventionists, they are generally not readily available to anesthesiologists or intensivists in perioperative or ICU settings when central venous cannulations are attempted.

In conclusion, although both of our cases ended without significant harm, physicians should be aware of risks associated with central venous cannulation especially with indwelling PICC or other endovascular devices to avoid preventable complications.

*Preventive Measures Include the Following*. Remove PICC prior to jugular or subclavian CVC, and consider feasibility of femoral CVC if anticipated as a bridge for short time and PICC is essential.

Left internal jugular cannulation may be technically easier in patients with right upper extremity catheters and vice versa.

Avoid forcing guidewire if met with resistance while advancing or retrieving and refrain from advancing guidewire more than 20cm in adult patient [12, 14].

To have straight tip or angle tip guidewire standby when attempting CVC in patients with indwelling endovascular devices of lines.

Physicians should be aware of PICC risk profile when deciding on central venous access which according to recent data is not very different from traditional CVC contrary to popular perception [15].
